# Irreducible Distal Tibia Physeal Injury with Tibialis Posterior Tendon Interposition

**DOI:** 10.1155/2021/6646953

**Published:** 2021-04-27

**Authors:** Chintan Doshi, Simran Dua, Shital N. Parikh

**Affiliations:** ^1^Division of Pediatric Orthopaedics, Cincinnati Children's Hospital Medical Center, 3333 Burnet Avenue, Cincinnati, OH 45229, USA; ^2^Lambert High School, 805 Nichols Rd, Suwanee, GA 30024, USA; ^3^Division of Pediatric Orthopaedics, Cincinnati Children's Hospital Medical Center, 3333 Burnet Avenue, Cincinnati, OH 45229, USA

## Abstract

A 14-year-old basketball player presented with a displaced distal tibia physeal fracture which is typically treated with closed reduction with or without internal fixation. However, repeated attempts at closed reduction failed to align the fracture fragments. At open reduction, tibialis posterior tendon interposition was identified within the fracture site and bowstringing of the tendon prevented closed reduction. A tendon interposition should be suspected when repeated closed reduction attempts fail to achieve satisfactory fracture reduction. The features of tendon interposition should be differentiated from the more common periosteal interposition for physeal fractures of the tibia.

## 1. Introduction

Physeal fractures of the distal tibia and the fibula are the most common physeal fractures of the lower extremity [1]. For displaced, extra-articular, physeal fractures of the distal tibia, i.e., Salter-Harris (SH) type I and II fractures, an initial attempt of closed reduction and immobilization is the recommended treatment. As with any physeal fracture, closed reduction methods should include sustained traction and gentle manipulation, to avoid any further physeal insult.

The most common soft tissue interposition between the physeal fracture fragments that would preclude an anatomic reduction is the periosteum [2]. Periosteal interposition in the physeal fracture site would manifest as a residual gap or physeal widening of 3 mm or more on radiographs [3]. It is controversial if removal of the periosteum from the physeal fracture site in SH types I and II fractures of the distal tibia would be of any benefit as studies on the rate of premature physeal closure have shown conflicting results [3, 4].

Very rarely, like in the described case report, the fracture is locked in a displaced position and it is irreducible by closed methods of traction and manipulation. In such instances, tendon interposition at the physeal fracture site should be suspected.

## 2. Case Presentation

**Figure 1:**
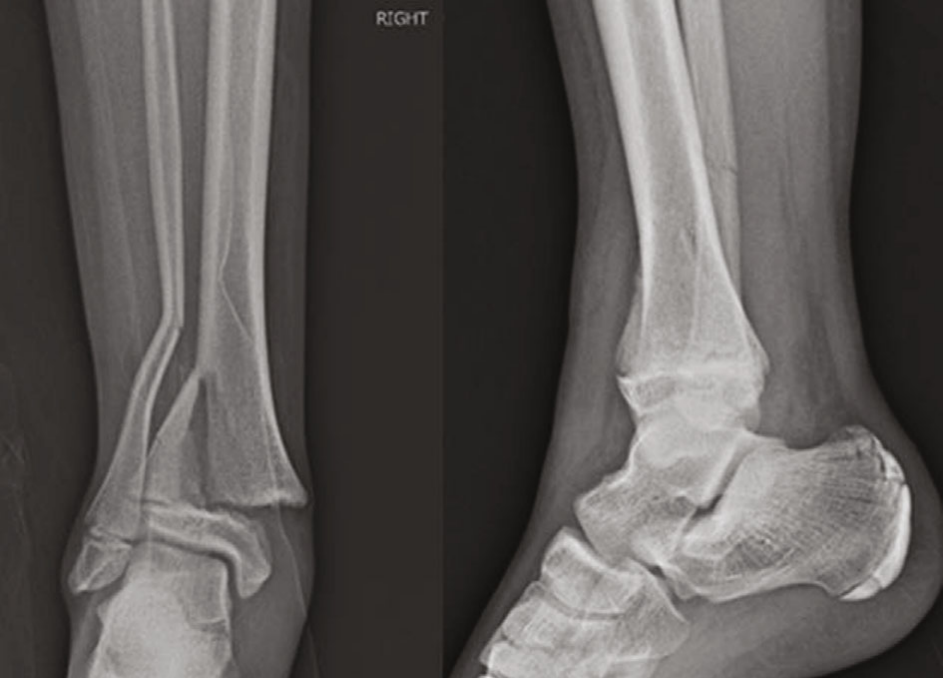
Anteroposterior and lateral radiographs of the ankle fracture at time of presentation.

A 14-year-old boy presented to the emergency department (ED) after sustaining a twisting injury to his right ankle while playing basketball. Clinical evaluation showed significant swelling and deformity around the ankle. There were no neurovascular deficits. He was unable to bear weight. Radiographs showed a displaced distal tibia SH type II physeal fracture and a transverse fracture of the fibula with valgus angulation (Figure 1).

**Figure 2:**
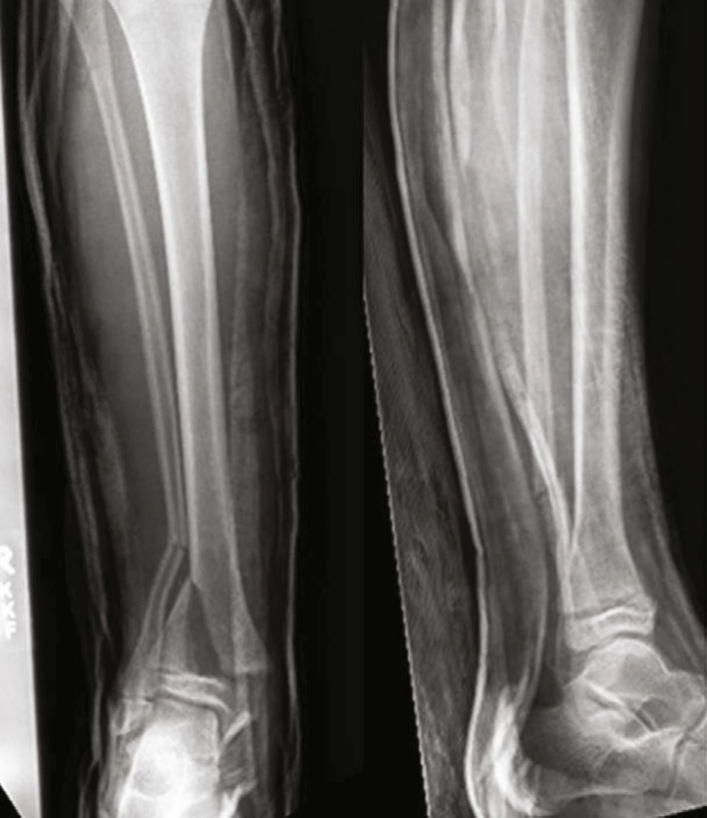
Anteroposterior and lateral radiographs of the ankle following attempt of closed redcution.

**Figure 3:**
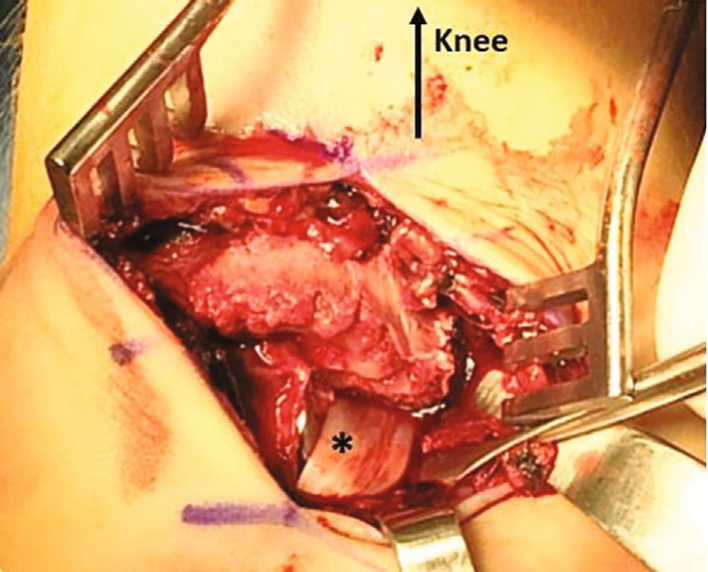
Intraoperative photo showing interposition of tibialis posterior tendon (^∗^) at the fracture site between the metaphysis and epiphysis.

**Figure 4:**
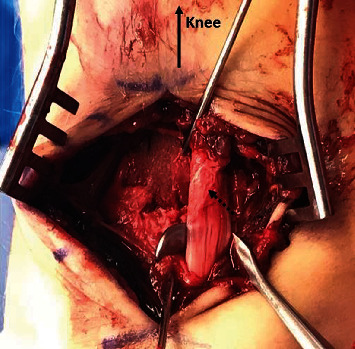
Mildly frayed tibialis posterior tendon (dashed arrow) repositioned and fracture reduction temporarily fixed by K-wire fixation.

**Figure 5:**
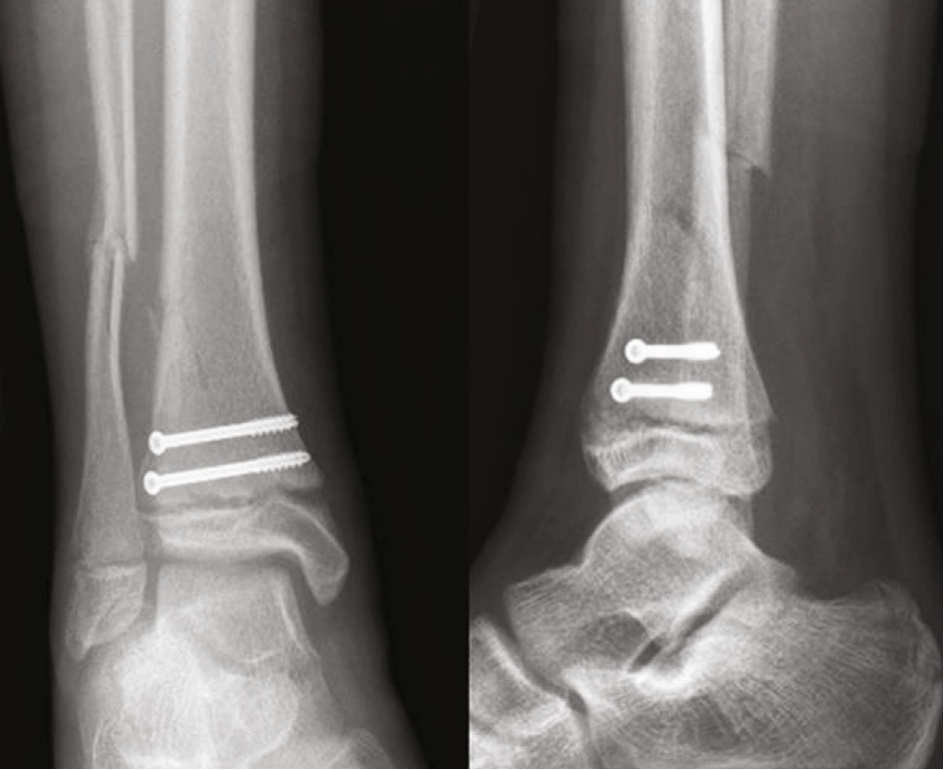
Postoperative radiograph in the anteroposterior and lateral views with fixation by screws.

An initial attempt at closed reduction performed in the ED under conscious sedation was unsuccessful; the fracture could not be moved from its displaced position after sustained traction and manipulation (Figure 2). The patient was taken to the operating room, and a second attempt at closed reduction was performed with the patient under general anesthesia and muscle relaxation. Again, the fracture could not be moved from its displaced position. Tendon interposition was suspected and open reduction of the fracture was performed by making an incision on the anteromedial aspect of the distal tibia centered over the metaphyseal spike. The fracture site was exposed, and the posterior tibial tendon was found interposed within the physeal fracture site. The tendon was bowstringing around the metaphyseal fragment from the proximal-posterior to distal-anterior direction between the metaphysis and epiphysis (Figure 3). Traction on the distal fragment increased the tension and worsened the bowstringing of the tendon. Hence, the traction was released and the interposed tendon was retracted out from the fracture site. This allowed the fracture to be reduced without difficulty. The tendon had mild fraying but did not show any laceration or tear (Figure 4). The fracture reduction was confirmed on fluoroscopic evaluation, and two 4 mm partially threaded cannulated screws were used for fracture compression and fixation across the large Thurston-Holland metaphyseal fragment (Figure 5).

**Figure 6:**
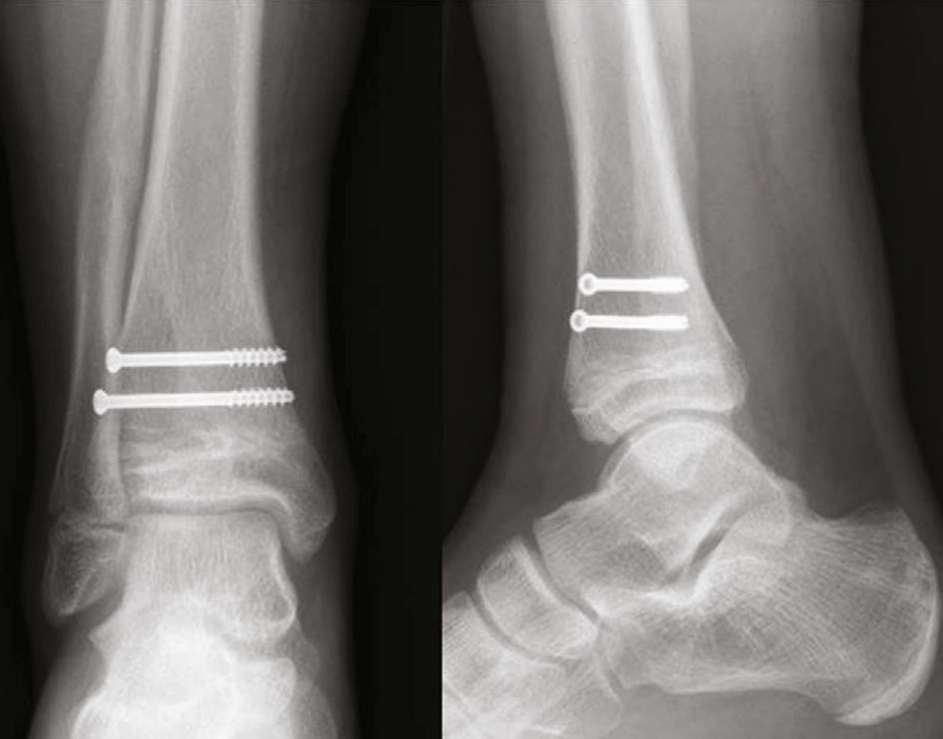
Anteroposterior and lateral radiographs of the ankle at 18-month follow-up.

Postoperatively, the leg was immobilized in a boot and nonweight bearing with crutches was used for 4 weeks, followed by weaning of the boot and gradual transition to full weight bearing. The patient was released to full activities at 4 months. Eighteen months postinjury, he had no pain or deformity, had full ankle range of motion, and had no limitations with sports. The radiographs showed complete healing of the fracture with no deformity and closing physis (Figure 6).

## 3. Discussion

SH type II fracture of the distal tibia is the commonest physeal fracture type, accounting for 32–40% of all distal tibia physeal fractures [5]. Besides the SH classification system, another classification system that is frequently used for ankle fractures in children is the Dias-Tachdjian classification, which is based on position of the foot and direction of the force at the time of injury [6]. The prognosis for this fracture is dependent on many factors like mechanism of injury, severity of injury, fracture type, degree of comminution, amount of displacement, number of reduction attempts, adequacy of reduction, fixation methods and remaining growth [5, 7]. There have been several reported cases of tibialis posterior tendon interposition in the ankle joint causing an irreducible ankle fracture-dislocation in adults. However, tibialis posterior tendon interposition in the physis is rare and seldom reported. Murakami et al. described an injury in an 11-year-old boy after a fall from height [8]. The tibial posterior tendon was interposed in the physeal fracture site and required open reduction, tendon extraction, and internal fixation with two K-wires. Soulier et al. described a similar injury in a 13-year-old soccer player following tackle injury [9]. Open reduction and internal fixation was performed using a cancellous screw and K-wires for tibia and plate fixation for the fibula. Both cases had no growth disturbances at follow-up.

Besides tibialis posterior tendon interposition, other soft tissue interpositions in the distal tibia physeal fracture site have been reported. In 1957, Johnson and Fahl reported a case of interposition of the anterior tibial tendon in the abduction type of epiphyseal displacement, which was not amenable to closed reduction, and an open reduction was required [10]. In 1983, Grace reported 3 cases of soft tissue interposition associated with SH type II fracture of the distal tibia: tibialis anterior tendon, extensor hallucis longus tendon, and the neurovascular bundle [11]. The concern for vascular compromise should be evaluated due to the close proximity of the neurovascular bundle to the tendinous structures which can interpose during the injury or during the attempt of closed reduction.

It is important to differentiate the more common periosteal interposition from the rare tendon interposition after attempts at closed reduction of the displaced distal tibial physeal fracture. Due to the tensile force at the time of injury, the periosteum would strip from the relatively weaker metaphyseal side attachment but would remain firmly adherent to the epiphysis; when these fracture fragments reduce, the stripped periosteum would be enfolded and entrapped in the physis. Clinically, despite periosteal interposition, closed reduction would correct the deformity and an anatomic or near-anatomic ankle alignment would be achieved. With tendon interposition, deformity remains persistent even after typical closed reduction maneuvers of traction and manipulation. With traction, the bowstringing of the tendon would worsen and fracture reduction would be increasingly difficult. Radiographically, periosteal interposition would present as residual gap or physeal widening of 3 mm or more but the fracture fragments are typically well-aligned. In contrast, with tendon interposition, the fracture fragments would not be well-aligned and step-off between fracture fragments would be persistent. For treatment, multiple studies have failed to show any significant advantage of open reduction to remove periosteal interposition. For tendon interposition, open reduction is mandatory to achieve fracture reduction. Once fracture reduction is obtained, internal fixation could be performed and satisfactory outcome could be expected.

In conclusion, for an irreducible distal tibial physeal fracture, tendon interposition should be suspected. Multiple attempts at closed reduction should be avoided as it can lead to tendon tear or injury to the nearby neurovascular structures. Open reduction, removal of interposed tissue, and internal fixation would allow for a satisfactory and safe reduction.

## Data Availability

Data was collected retrospectively from Cincinnati Children's Hospital and Medical Center patients through an IRB exempt research study. Cincinnati Children's IRB does not allow patient data to be deposited in unaffiliated third party registries.
